# Investigation of the acaricidal activity of the acetone and ethanol extracts of 12 South African plants against the adult ticks of *Rhipicephalus turanicus*

**DOI:** 10.4102/ojvr.v84i1.1523

**Published:** 2017-11-23

**Authors:** Gerda Fouche, Bellonah M. Sakong, Olubukola T. Adenubi, Jean Paul Dzoyem, Vinny Naidoo, Tlabo Leboho, Kevin W. Wellington, Jacobus N. Eloff

**Affiliations:** 1 Council for Scientific and Industrial Research (CSIR) Biosciences, Pretoria, South Africa; 2 Department of Paraclinical Sciences, University of Pretoria, South Africa; 3 Biomedical Research Center, University of Pretoria, South Africa

## Abstract

The acaricidal activity of acetone and ethanol extracts of 12 plant species was evaluated using the contact method on *Rhipicephalus turanicus* (Acari: Ixodidae) ticks at an initial concentration of 20% (200 mg/mL). Eight of the 12 plants had mortality greater than 50% and the acetone extracts had better acaricidal activity than the ethanol extracts. The acetone extract of *Calpurnia aurea* (leaves and flowers) had the highest corrected mortality (CM) of 92.2% followed by *Schkuhria pinnata* (whole plant) with a CM of 88.9%, *Ficus sycomorus* (bark and stems) 86.7% and *Senna italica* subsp. *arachoides* (roots, leaves and fruits) 83.3%. Selected extracts were tested at five different concentrations using the adult immersion test. From dose–response assays, EC_50_ values of 61.82 mg/mL, 115.21 mg/mL and 161.02 mg/mL were obtained for the acetone extracts of *S. pinnata* (whole plant), *S. italica* subsp. *arachoides* (roots, leaves and fruits) and *C. aurea* (leaves and flowers) respectively. The ethanol extract of *Monsonia angustifolia* (whole plant) had the highest CM of 97.8% followed by *S. pinnata* (whole plant) with a CM of 86.7%, *C. aurea* (leaves and flowers) 81.1% and *Cleome gynandra* (leaves) 77.8%. There is potential for the development of environmentally benign botanicals as natural acaricides against *R. turanicus*.

## Introduction

Globally, approximately 80% of 1.2 billion cattle are at risk of ticks and tick-borne diseases which account for a global annual loss of $ 7 billion (Bagavan et al. 2009; Zahir et al. 2010). Ticks are vectors of various pathogens such as bacteria, viruses and protozoa (Boldbaatar et al. 2006; Kocan, Blouin & De la Fuente 2011). When livestock are parasitised by ticks, the quality of animal products is reduced and livestock death may also occur (Elango & Rahuman 2011; Tian et al. 2011). The tick species, *Rhipicephalus turanicus*, is broadly dispersed throughout the world and has been documented in several parts of Africa, Asia and Europe (Filippova 1994; Morel & Vassiliades 1962; Pegram et al. 1987; Pomerantsev 1948). It is also commonly found in Mediterranean countries (Feldman-Muhsam & Saturen 1961; Gilot et al. 1992; Morel & Vassiliades 1962; Mumcuoglu et al. 1993). This tick parasitises a wide range of hosts which includes humans and dogs. It is a vector of rickettsial diseases and thus of medical and veterinary importance. *Rhipicephalus turanicus* is a vector of North Asian tick typhus and Q-fever caused by *Rickettsia sibirica* (Balashov & Daiter 1973; Berdyev 1980).

Some common anti-tick methods that have been used include applying synthetic acaricides both to the environment and to animals, the spraying of synthetic drugs, using smoke agents in forests as acaricidal drugs and consistent medicated bathing of livestock (Iori et al. 2005; Patarroyo et al. 2009; Regassa 2000). Tick control strategies are presently directed at averting production loss, dropping tick numbers to appropriate levels, decreasing chemical residue risks and reducing the dependence on chemicals by exploiting different control treatments for different herd groups (Ghosh, Azhahianambi & Yadav 2007).

A viable alternative treatment to the use of synthetic compounds for controlling tick infestations in livestock is phytotherapy (Madzimure et al. 2011; Moyo & Masika 2009). Advantages of using botanical extracts are that they are biodegradable, less toxic to the environment and to non-targeted species (Liang, Chen & Liu 2003) when compared with chemical agents that accumulate and pollute the environment. Other researchers have obtained promising results in controlling ticks by using botanicals (Fernandes & Paula Souza Freitas 2007). We therefore investigated several South African plants based on literature reports and ethno-veterinary use by traditional communities to control ticks in livestock. We have previously reported on the acaricidal activity of organic extracts of South African plants against the larvae of both *Rhipicephalus decoloratus* (Koch 1844) (Acari: Ixodidae) (Fouche et al. 2016a) and *Rhipicephalus* (*Boophilus*) *microplus* (Wellington et al. 2016) as well as their anthelmintic activity against *Haemonchus contortus* (Fouche et al. 2016b). These results prompted us to determine whether the organic plant extracts would also have acaricidal activity against adult ticks of *R. turanicus* and report here on the results of our study.

## Materials and methods

### Plant material collection and preparation

The plants collected and the methods followed were as described by Fouche et al. (2016a).

### Ticks

Adult stages of *R. turanicus* ticks (both sexes) were obtained from Clinvet International, Bloemfontein, South Africa. The ticks were kept at the Phytomedicine Laboratory, Department of Paraclinical Sciences, Faculty of Veterinary Sciences, University of Pretoria, in glass humidity chambers closed by a removable cover at an average temperature of 25 °C (± 1 °C). Relative humidity of 75% ± 10% was maintained by placing saturated sodium chloride solution in the glass chamber. The ticks were stored in vials covered with cotton mesh (to allow normal air exchange) and set on a square glass plate placed at the base of the chamber on four small bearings, so that the edges of the plate were at a distance of 1.5 cm from the walls. In this way, the saturated saline solution on the floor also prevented the ticks from reaching the walls.

### Determination of the acaricidal activity using an *in vitro* tick toxicity bioassay

The contact bioassay described by Zorloni, Penzhorn and Eloff (2010) was employed. One microlitre of a 20% (200 mg/mL) concentration of each extract was dropped on the dorsum of each tick (*n* = 10) and left for 1 min before storing them in a vial covered with a perforated stopper. The procedure was followed for the negative control (acetone or distilled water) and the positive control (cypermethrin 5 mg/mL). Each treatment was repeated at three different times. Mortality was determined 24 h after exposure by examination with a stereo microscope. Ticks were recorded as alive if they exhibited normal behaviour on exposure to the CO_2_ in human breath or when physically stimulated with a plastic tweezer. Ticks were confirmed dead based on signs of cuticle darkness, stopped Malpighian tube movement and haemorrhagic skin lesions. Those showing some difficulty in movement or maintaining a normal posture were termed weak or very weak if there was no leg coordination or ability to right themselves. The percentage of mortality in all the experimental groups was corrected by applying Abbott’s formula (Abbott 1987):

$$\text{Corrected percent mortality(\%)}=\frac{\%\text{test mortality}-\%\text{control mortality}}{100-\%\text{control mortality}}\times 100$$ [Eqn 1]

### Dose–response bioassay

Based on preliminary screening results (> 80% efficacy), two-fold graded decreasing concentrations (from 20% to 1.25%) of acetone extracts of *Calpurnia aurea, Schkuhria pinnata* and *Senna italica* were used. Ten adult ticks were immersed in the test concentrations for 1 min; test solutions were decanted, treated ticks were dried over filter papers and kept at 28 °C and 85% RH in 20 mL glass vials closed with a perforated stopper for air exchange as described by Al-Rajhy et al. (2003). Graded two-fold decreasing concentrations of cypermethrin served as the positive control. Each treatment was also repeated at three different times and mortality rate was recorded after 24 h.

### Determination of the toxicity of the plant extracts

The toxicity of the plant extracts was previously determined using the MTT [3-(4,5-dimethylthiazol-2-yl)-2,5-diphenyltetrazolium bromide] assay as described by Fouche et al. (2016a). A plant extract has an acceptable level of toxicity when the LC_50_ value exceeds 20 μg/mL but a lower value is regarded as toxic. The results have been included in Table 1 for convenience.

**TABLE 1:** The mortality, corrected mortality and concentration in mg/mL killing of 50% of Vero cells (LC_50_) of the 12 indigenous South African plant species screened against adult ticks of *Rhipicephalus turanicus*.

Entry	Plant and plant part used in extraction	Solvent	Mortality (%)	CM (%)	Vero cells LC_50_ (µg/mL)
1	*Aloe rupestris* (leaves)	Acetone	0	0.0	63.46 ± 11.00
		Ethanol	20	11.1	101.99 ± 3.86
2	*Calpurnia aurea* (leaves and flowers)	Acetone	**93**	**92.2**	166.63 ± 7.97
		Ethanol	**83**	**81.1**	504.32 ± 3.90
3	*Senna italica* (roots, leaves and fruits)	Acetone	**85**	**83.3**	46.31 ± 2.89
		Ethanol	**83**	**81.1**	550.67 ± 12.49
4	*Cissus quadrangularis* (stems)	Acetone	16	6.67	41.44 ± 2.96
		Ethanol	20	11.1	74.33 ± 3.68
5	*Clematis brachiata* (whole plant)	Acetone	**60**	**55.6**	117.00 ± 4.08
		Ethanol	**53**	**47.8**	485.28 ± 21.74
6	*Cleome gynandra* (leaves)	Acetone	**70**	**66.7**	553.61 ± 18.83
		Ethanol	**80**	**77.8**	39.51 ± 0.36
7	*Ficus sycomorus* (bark and stems)	Acetone	**88**	**86.7**	172.94 ± 8.91
		Ethanol	23	14.4	458.36 ± 7.87
8	*Monsonia angustifolia* (whole plant)	Acetone	15	5.6	120.37 ± 4.06
		Ethanol	**98**	**97.8**	34.67 ± 0.86
9	*Pelargonium luridum* (whole plant)	Acetone	45	38.9	30.58 ± 3.40
		Ethanol	45	38.9	32.86 ± 1.06
10	*Schkuhria pinnata* (whole plant)	Acetone	**90**	**88.9**	39.93 ± 1.80
		Ethanol	**88**	**86.7**	89.14 ± 4.14
11	*Sclerocarya birrea* (bark and root)	Acetone	0	0.0	418.27 ± 7.89
		Ethanol	33	25.6	486.71 ± 3.11
12	*Tabernaemontana elegans* (leaves)	Acetone	0	0.0	32.35 ± 0.88
		Ethanol	13	3.3	40.04 ± 4.78
13	Doxorubicin	-	-	-	2.97 ± 0.016
14	Negative control (acetone only)	-	10	-	ND
15	Negative control (water)	-	0	-	ND
16	Positive control (Cypermethrin)	-	100	-	ND

Mortality in the range 50% – 100% are in bold.

CM, corrected mortality; ND, not determined.

### Statistical analysis

Data on percentage mortality were subjected to a one-way analysis of variance (ANOVA) for comparison. Mean per cent adult mortality, cytotoxicity and their associated confidence intervals were estimated using Bonferroni’s and Dunnett’s Multiple Comparison Tests on GraphPad Prism 5.0 for windows (GraphPad Software 2013, Inc., USA). Lethal concentrations at 50% and slope levels were considered significantly different if their associated confidence intervals did not overlap. All differences were considered significant if *p* ≤ 0.05.

## Results

### Determination of the acaricidal activity

**FIGURE 1:**
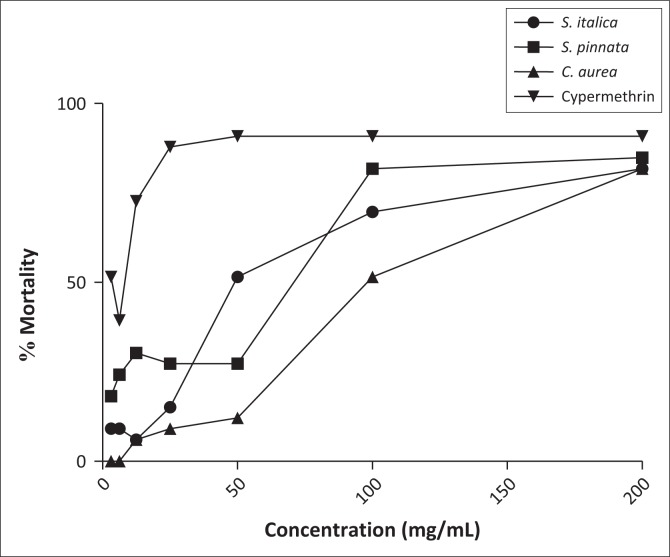
The dose-dependent mortality of the acetone extracts against *Rhipicephalus turanicus*.

The plant extracts were exposed *in vitro* to adult ticks of *R. turanicus* for efficacy testing. Cypermethrin, a synthetic chemical based on the pyrethrins in pyrethrum extract which comes from the chrysanthemum plant (NPIC), was used as a positive control. The results are shown in Table 1. Dose–response assays for determining the EC_50_ values of the extracts after 24 h treatment on the ticks were conducted (Table 2). This mortality was then used to determine the EC_50_, the concentration that killed 50% of the ticks. Values found were 61.82 mg/mL, 115.21 mg/mL and 161.02 mg/mL for the acetone extracts of *S. pinnata* (whole plant), *S. italica* (roots, leaves and fruits) and *C. aurea* (leaves and flowers), respectively (Table 3). The dose-dependent mortality of the acetone extracts against *R. turanicus* can be seen in Figure 1.

**TABLE 2:** Dose-dependent mortality of the acetone extracts against adult ticks of *Rhipicephalus turanicus*.

Entry	Plant and plant part used in extraction	Concentration (mg/mL)	Mortality (%)
1	*Senna italica* (roots, leaves and fruits)	100.0	77
		50.0	57
		25.0	17
		12.5	7
2	*Schkuhria pinnata* (whole plant)	100.0	90
		50.0	30
		25.0	30
		12.5	33
3	*Calpurnia aurea* (leaves and flowers)	100.0	57
		50.0	13
		25.0	10
		12.5	7
4	Cypermethrin	5.5	100
		2.75	100
		1.375	100
		0.6875	97

**TABLE 3:** The EC_50_ values of selected acetone extracts.

Entry	Plant and plant part used in extraction	EC_50_ (mg/mL) acetone extract
1	*Schkuhria pinnata* (whole plant)	61.82
2	*Senna italica* (roots, leaves and fruits)	115.21
3	*Calpurnia aurea* (leaves and flowers)	161.02
4	Cypermethrin	0.25

### Acaricidal activity of the acetone extracts

The acetone extracts were generally not soluble in water and acetone and led to the death of about 10% of the ticks. The best acaricidal activity (92.2%) was observed for the leaf and flower extracts of *C. aurea* followed by the leaf extract of *S. pinnata* (88.9%), the bark and stem extracts of *Ficus sycomorus* (86.7%) and the root, leaf and fruit extracts of *S. italica* subsp. *arachoides* (83.3%). The leaf extract of *Cleome gynandra* had a mortality of 66.7%, whereas it was 55.6% for the whole plant extract of *Clematis brachiata*.

### Acaricidal activity of the ethanol extracts

The ethanol extracts generally had lower mortality than that of the acetone extracts. The best acaricidal activity (97.8%) was observed for the whole plant extract of *Monsonia angustifolia* which also had the highest mortality of both the ethanol and acetone extracts. This was followed by the leaf extract of *S. pinnata* (86.7%), the root, leaf and fruit extracts of *S. italica* subsp. *arachoides* (81.1%) and the leaf extract of *C. gynandra* (77.8%).

### Determination of the toxicity of the plant extracts

The toxicity of the plant extracts against Vero cells was determined in a previous study and was found to be acceptable (Fouche et al. 2016a).

## Discussion

From the results of the efficacy testing against *R. turanicus*, it is evident that 8 of the 12 plants had some acaricidal activity (Table 2) at a 20% (w/v) concentration. Other workers have also evaluated the acaricidal effects of plants at that concentration (Cetin et al. 2009; Godara et al. 2014; Vendramini et al. 2012; Zorloni et al. 2010) while others have used higher concentrations such as 250 mg/mL and higher (Coskun et al. 2008; Domingos et al. 2013; Kongkiatpaiboon et al. 2014). The concentrations used in tests should, however, not exceed realistic levels in field trials (Schmeda-Hirschmann & De Arias 1992). On average, the acetone extracts had a higher mortality than the ethanol extracts but the highest mortality was observed for the ethanol extract from *M. angustifolia* (leaves).

The acetone extract of *C. aurea* (leaves and flowers) had 92.2% mortality while the ethanol extract had 81.1% mortality against *R. turanicus*. Similarly, Zorloni et al. (2010) reported that 20% and 10% acetone leaf extracts for *C. aurea* (leaves) either kill or severely compromise the movement of unfed adult *Rhipicephalus pulchellus* ticks. In another study, it was proposed that *C. aurea* extracts can probably be used as baits in a trap for the management of ticks in the field (Nana et al. 2010). According to Adedapo et al. (2008), phenolic compounds are the major chemical components of *C. aurea* which are accountable for the attraction behaviour of over 12 species of ixodid ticks (McDowell & Wallade 1986; Wood et al. 1975; Yoder & Stevens 2000). The efficacy of the ethanol leaf extract of *C. aurea* may be attributed to its ability to attract and also kill or compromise the movement of ticks. Comparable activities were found in *C. aurea* collected from Ethiopia despite the plant in South Africa growing under widely diverse environmental conditions. A total of 28 plant species, used to control ticks on animals in southern Ethiopia, were evaluated and were found to have good acaricidal activity (Zorloni 2008).

The acetone extract of *S. italica* subsp. *arachoides* (roots, leaves and fruits) had a slightly higher mortality (83.3%) than the ethanol extract (81.1%). The root extracts of *S. italica* subsp. *arachoides* had acaricidal activity against adults of *Hyalomma marginatum rufipes* (Magano et al. 2008) with the ethyl acetate extract being the most potent among several extracts (hexane, chloroform, dichloromethane, ethyl acetate and methanol) tested. The ethyl acetate extract was found to contain 1,2-benzenedicarboxylic acid, dibutyl ester, 1,8-dihydroxy-3-methylanthraquinone, 1,2-benzenedicarboxylic acid, bis(2-ethylhexyl) ester, hexadecanoic acid, 9-hexadecanoic acid as chemical components. Flavonoids, tamarixetin (3-rutinoside-7-rhamnoside), *ß*-sitosterol, stigmasterol, α-amyrin, 1,5-dihydroxy-3-methylanthraquinone and anthraquinone were isolated from the aerial parts of *S. italica* subsp. *arachoides* (Elsayed et al. 1992). Compounds such as β-sitosterol (1), (22*E*)-3-β*-*hydroxycycloart-22-en-24-one, uvaol, daucosterol, methyl-3,4-dihydroxybenzoate, emodin, 4-hydroxyphenyl-*O*-β-D-glucopyranoside, aloin B and rutin were isolated from the methanol extract of the aerial parts (Asfour, Ibrahim & Mohamed 2015). Because of the similar polarity of methanol and ethanol, it is likely that these compounds were also extracted with ethanol and would thus be present in the ethanol extract in this present study.

The acetone extract of *C. brachiata* (whole plant) was more lethal (55.6%) than its ethanol extract (47.8%). Okalebo (2002) found that the leaves, stem and roots tested positive for anthraquinones, alkaloids, saponins, coumarins, sterols, carotenoids and flavonoids, and cardenolides. Tannins were only present in the stem and leaves. The root had the highest amounts of alkaloids and anthraquinones. In another study investigating the antimicrobial activity of this plant species, phytochemical screening revealed the presence of phenols, tannins, saponins, flavonoids, terpenoids and glycosidic compounds in the methanol and acetone leaf extracts (Mostafa & Afolayan 2013). Thus, these compounds may be responsible for the observed acaricidal activity of the acetone extract of *C. brachiata* (whole plant).

Neither the acetone nor ethanol stem extracts of *Cissus quadrangularis* (stem) had notable mortality (6.67% and 11.1%, respectively) against *R. turanicus.* Santhoshkumar et al. (2012) did, however, find that the aqueous extract of *C. quadrangularis* (stem) had acaricidal activity against *R*. (*B*.) *microplus*. Silver nanoparticles were also prepared from the aqueous stem extract and were found to be more active against *R*. (*B*.) *microplus* than the aqueous stem extract.

The acetone extract of *C. gynandra* (leaves) led to a mortality of 66.7% and the ethanol extract a mortality of 77.8% against *R. turanicus*. These results correlate with that reported by Malonza et al. (1992) who also reported on the acaricidal activity of the leaves of this plant. High levels of mortality against nymphal *Amblyomma variegatum* and *Rhipicephalus appendiculatus* were observed. All *R. appendiculatus* nymphs died within 6–16 h, but only 71% of *A. variegatum* nymphs died after 2 h of continuous exposure to the plant leaves (Malonza et al. 1992). In a phytochemical analysis study on the leaves, ethanol extracts showed more phytochemicals than acetone. Based on spot tests, tannins, phenols, flavonoids, cardiac glycosides, steroids, saponins and alkaloids were found to be present (Srinivas et al. 2014). The acaricidal activity of the acetone and the ethanol extracts of *C. gynandra* (leaves) may thus be attributed to the presence of these compounds.

The acetone extract of *F. sycomorus* (bark and stems) led to a mortality of 86.7% while the ethanol extract had low mortality (14.4%). Phytochemical screening of the n-hexane chloroform, ethyl acetate, n-butanol and water fractions found that flavonoids, coumarins, quninous, alkaloids, triterpenes, steroids and saponins are present in all the fractions (Osama & Awdelkarim 2015). These compounds are likely to be present in the acetone extract having acaricidal activity.

The acetone extract of *M. angustifolia* (whole plant) had no notable mortality (5.6%) while the ethanol extract had excellent mortality (97.8%) almost as potent as cypermethrin (100%). Khorombi (2006) isolated five aryl naphthalene lignans (suchilactone, justicidin A, 5*-*methoxyjusticidin A, chinensinaphthol and retrochinensinaphthol methyl ether) during the fractionation of the organic (methanol-dichloromethane) extract of *M. angustifolia*. Podophyllotoxin, a naturally occurring aryltetralin lignan, has interesting insecticidal and antifungal activities (Wang et al. 2014). The lignans isolated from *M. angustifolia* (whole plant) may thus be responsible or may have contributed to the acaricidal activity in this present study.

For *S. pinnata* (whole plant), high mortality was observed for both the acetone and ethanol extracts (88.9% and 86.7%, respectively). The plant chemicals that have thus far been identified in *S. pinnata* are chromolaenide, chromolaenolide, costunolides, dithiin, eucannabinolides, germacranolides, heliangolides, hiyodorilactones, loliolide, nerols, pectolinarigenin, santhemoidin A, schkuhrianol, schkuhrins, schkuhripinnatolides, schkurianol, thiarubrine A, thiophene, tridecapentayne and zaluzanin C (Rain-tree). In a study by Rodrigo (2010) on the antiproliferative activity, it was found that the ethanol extracts of the leaves contained sterols and/or triterpenes and flavonoids. These compounds may be responsible for the observed acaricidal activity by *S. pinnata* (whole plant).

For *Aloe rupestris* (leaves), *Pelargonium luridum* (whole plant), *Sclerocarya birrea* (bark, root) and *Tabernaemontana elegans* (leaves), both the acetone and ethanol extracts had low mortality (mortality < 50%).

The toxicity of the plant extracts against Vero cells was determined in a previous study in which it was found that it was acceptable (> 20 µg/mL) and none were as toxic as the positive control, doxorubicin (2.97 µg/mL ± 0.016 µg/mL).

From the data in Table 2 a clear dose-related effect was established. The EC_50_ values were calculated to be 61.82 mg/mL, 115.21 mg/mL and 161.02 mg/mL for the acetone extracts of *S. pinnata* (whole plant), *S. italica* (roots, leaves and fruits) and *C. aurea* (leaves and flowers), respectively (Table 3).

Of the plant species investigated in the present study, the leaves were the most used plant part traditionally followed by the whole plant. It was also found that leaves were the most used plant part in an ethnobotanical survey of the lowlands of the Konta people of Ethiopia.

The pharmaceutical industry continues to investigate the potential of natural products as sources of novel medicinal compounds (Ghosh & Playford 2003). An estimated 14% – 28% of higher plant species are used medicinally and 74% of pharmacologically active plant-derived components were discovered based on the ethnomedicinal or ethno-veterinary use of the plants (Eloff 1998). In this study, the use of ethno-veterinary leads for selecting plants for screening has increased the probability of discovering novel, natural acaricides for combatting *R. turanius* and other tick species.

## Conclusion

*Monsonia angustifolia, C. aurea and S. pinnata* have potent acaricidal activity against *R. turanicus*. The plants used in this study have low toxicity against Vero cells. These results show that plant extracts have potential as acaricidal agents against *R. turanicus* and validate that a botanical acaricide can also be effective in tick management instead of chemical acaricides. If the safety can be established in animal trials, some of these extracts may have sufficiently high activity to be considered as a treatment in rural areas. The isolation of compounds responsible for the acaricidal activity in these extracts could lead to the discovery of novel natural acaricidal agents that are less toxic to the environment and non-targeted species.
