# Corrigendum: Investigation of the acaricidal activity of the acetone and ethanol extracts of 12 South African plants against the adult ticks of *Rhipicephalus turanicus*

**DOI:** 10.4102/ojvr.v88i1.1950

**Published:** 2021-07-27

**Authors:** Gerda Fouche, Bellonah M. Sakong, Olubukola T. Adenubi, Jean Paul Dzoyem, Vinny Naidoo, Tlabo Leboho, Mbokota C. Khosa, Kevin W. Wellington, Jacobus N. Eloff

**Affiliations:** 1 Council for Scientific and Industrial Research (CSIR) Biosciences, Pretoria, South Africa; 2 Department of Paraclinical Sciences, University of Pretoria, South Africa; 3 Biomedical Research Center, University of Pretoria, South Africa; 4 Agricultural Research Council – Tropical and Subtropical Crops, Nelspruit, South Africa

In the published version of this article, Fouche, G., Sakong, B.M., Adenubi, O.T., Dzoyem, J.P., Naidoo, V., Leboho, T. et al., 2017, ‘Investigation of the acaricidal activity of the acetone and ethanol extracts of 12 South African plants against the adult ticks of *Rhipicephalus turanicus*’, *Onderstepoort Journal of Veterinary Research* 84(1), a1523. https://doi.org/10.4102/ojvr.v84i1.1523, the seventh author, Mbokota C. Khosa, was omitted from the ‘Authors’ and ‘Affiliations’ sections. The indicated author should be added as the seventh author, and the following affiliation should be added as his affiliation: Agricultural Research Council – Tropical and Subtropical Crops, Nelspruit, South Africa.

The Authors’ contributions section is hereby update to:

## Authors’ contributions

G.F. conceptualised the study. M.C.K. was involved in the collection of some of the plant material and in the preparation of the extracts used in the biological screening assays. G.F., K.W.W. and T.L. carried out the literature search and plant selection. T.L. prepared the plant extracts. J.N.E. conceptualised the study in a joint application and supervised the students and postdoctoral fellows. V.N. supervised determination of acaricidal activity. J.P.D. supervised the determination of cytotoxicity. B.M.S. determined vero cell toxicity. M.C.K. was also involved in the fractionation and isolation process in the natural product chemistry laboratory. O.T.A. determined the acaricidal activity against adult ticks of *R. turanicus*. K.W.W. wrote the first draft of the manuscript.

